# CYP1B1 promotes angiogenesis and sunitinib resistance in clear cell renal cell carcinoma via USP5-mediated HIF2α deubiquitination

**DOI:** 10.1016/j.neo.2025.101186

**Published:** 2025-05-27

**Authors:** Ke Ma, Qinyu Li, Yi Zhang, Jiuyi Wang, Wei Jia, Jihong Liu, Bo Liu, Qiang Li, Qinzhang Wang, Kai Zeng

**Affiliations:** a Department of Urology, the First Affiliated Hospital of Shihezi University, Shihezi, Xinjiang, China; b Department of Oncology, Tongji Hospital, Tongji Medical College, Huazhong University of Science and Technology, Wuhan, Hubei, China; c Department of Pathology, the First Affiliated Hospital of Shihezi University, Shihezi, Xinjiang, China; d Department of Urology, Tongji Hospital, Tongji Medical College, Huazhong University of Science and Technology, Wuhan, Hubei, China

**Keywords:** Angiogenesis, Clear cell renal cell carcinoma, HIF2α, Sunitinib resistance, Ubiquitination

## Abstract

Clear cell renal cell carcinoma (ccRCC) is strongly aetiologically associated with von Hippel‒Lindau (VHL) tumour suppressor gene mutations, which result in constitutive activation of hypoxia-inducible factors and pathological angiogenesis. Although accumulating evidence indicates that antiangiogenic therapies targeting VEGF signalling can prolong the survival of ccRCC patients, the frequent development of therapeutic resistance to tyrosine kinase inhibitors such as sunitinib remains a critical clinical limitation. Through integrated multiomics analyses of sunitinib-resistant cell models, patient-derived xenografts, and clinical specimens, we systematically identified CYP1B1 as a central mediator of treatment resistance. Transcriptomic and genomic profiling revealed that CYP1B1 overexpression in resistant tumours functionally contributes to enhanced angiogenic potential and maintenance of the resistant phenotype. Mechanistic investigations demonstrated that CYP1B1 stabilizes hypoxia-inducible factor 2α (HIF2α) by facilitating USP5-mediated deubiquitination, thereby preventing proteasomal degradation. Notably, we identified VHL as a novel E3 ubiquitin ligase that regulates CYP1B1 turnover; notably, VHL deficiency in ccRCC promotes CYP1B1 protein accumulation by suppressing ubiquitination. These findings establish a feed-forward regulatory axis in which VHL loss-induced CYP1B1 stabilization promotes HIF2α signalling persistence, ultimately driving sunitinib resistance. Our study delineated the CYP1B1-USP5-HIF2α signalling cascade as a critical resistance mechanism and thus reveals a targetable vulnerability in treatment-refractory ccRCC.

## Introduction

Renal cell carcinoma (RCC) is one of the most prevalent malignancies worldwide [1]. ccRCC, the predominant subtype, is characterized primarily by inactivation of the VHL tumour suppressor gene, which leads to dysregulation of angiogenesis [2]. Aberrant vascularization plays a critical role in tumour growth and progression, profoundly influencing the tumour microenvironment and therapeutic response [3]. Tyrosine kinase inhibitors (TKIs), such as sunitinib, effectively suppress tumour angiogenesis and proliferation by targeting multiple angiogenic pathways [4,5]. Despite the significant extension of progression-free survival achieved with antiangiogenic therapy, many patients eventually develop resistance, leading to disease progression [6].

Recent studies have revealed that vasculogenic mimicry (VM) is a crucial mechanism underlying resistance to TKIs. Unlike conventional angiogenesis, which relies on endothelial cells to form blood vessels, VM enables tumour cells to generate vessel-like structures independently, ensuring a continued nutrient supply even when endothelium-dependent angiogenesis is inhibited by TKIs [[7], [8], [9]]. The disruption of conventional vascular networks by sunitinib is counteracted by tumour adaptation through VM, enabling sustained growth and invasion [10]. This adaptive mechanism facilitates tumour evasion from antiangiogenic therapy, ultimately contributing to resistance and disease persistence [11,12].

Cytochrome P450 (CYP) enzymes play pivotal roles in oncogenesis by converting procarcinogens into active carcinogens, thereby promoting tumour initiation and progression. Additionally, these enzymes are integral to the metabolism of chemotherapeutic agents and may decrease their efficacy [13,14]. Among the various CYP isoforms, CYP1B1 has garnered significant attention because of its distinctive expression profile and its role in cancer biology and drug resistance [15]. CYP1B1 may mediate chemoresistance by deactivating the polyene paclitaxel, thereby promoting cancer cell survival, stemness, and resistance to paclitaxel in lung cancer [16]. Moreover, the CYP1B1-mediated synthesis of 4‑hydroxy-oestradiol has been shown to contribute to castration resistance via oestrogen receptor α activation and IL-6 upregulation in prostate cancer [17]. Although CYP1B1 has been recognized as an oncogene in ccRCC [18], its role in conferring TKI resistance remains largely unexplored.

In this study, we utilized high-throughput sequencing data from sunitinib-resistant (SU-R) cell models, patient-derived xenografts (PDXs), and clinical samples to identify key genes contributing to sunitinib resistance. Our analysis revealed that CYP1B1 is significantly upregulated in SU-R cells and plays a pivotal role in promoting angiogenesis and sustaining sunitinib resistance. Mechanistically, CYP1B1 facilitates the deubiquitination of HIF2α via USP5, thereby stabilizing HIF2α. Additionally, we observed that CYP1B1 expression was markedly upregulated in VHL-deficient RCC, as pVHL mediates the ubiquitination and degradation of CYP1B1. Collectively, our findings reveal a previously unrecognized mechanism underlying sunitinib resistance in ccRCC and highlight CYP1B1 as a promising therapeutic target for overcoming resistance to TKIs.

## Materials and methods

### Cell lines and tissue samples

The 786O and ACHN cell lines were procured from the American Type Culture Collection (ATCC, Manassas, USA) and cultured in accordance with American Type Culture Collection (ATCC) guidelines. The OSRC2 cell line was acquired from ProCell (Wuhan, China) and maintained in RPMI-1640 medium enriched with 10 % FBS (Boster, Wuhan, China). To establish an SU-R RCC cell line (SU-R RCC), wild-type (WT) RCC cells were initially treated with 2 µM sunitinib (Selleck Chemicals, Munich, Germany), with the concentration increasing to 14 µM over a period of 16 weeks. Subsequently, SU-R RCC cells were maintained in culture media supplemented with 10 µM sunitinib. Human umbilical vein endothelial cells (HUVECs) were obtained from ScienCell (California, USA) and cultured according to the manufacturer’s guidelines. All the cell lines were authenticated as described above and maintained in a sterile incubator at 37°C with 5 % CO_2_.

Patients who were diagnosed with RCC and who underwent partial or radical nephrectomy at the First Affiliated Hospital of Shihezi University from July 2020 to March 2023 were enrolled, and tissues were collected. The clinical criterion for sunitinib resistance in patients was defined as disease progression during treatment, according to the Response Evaluation Criteria in Solid Tumors (RECIST) [19]. Progressive disease was characterized by *a* ≥ 20 % increase in the sum of the longest diameters of target lesions or the emergence of new lesions. Ethical approval for this study was granted by the Ethics Committee of the First Affiliated Hospital of Shihezi University (KJ2023-296-01). Informed consent was obtained from all participants, who consented to the utilization of their tissue samples for scientific investigation.

### Plasmid transfection

For gene knockdown, short hairpin RNA (shRNA) and nontarget shRNA were designed and synthesized by Genomeditech (Shanghai, China). The specific sequences used were as follows: sh-CYP1B1: sh-1#: 5′-GCATGATGCGCAACTTCTTCA-3′; sh-2#: 5′-GCAACTTCAGCAACTTCATCC-3′; and sh-3#: 5′-CAGCATGATGCGCAACTTCTT-3′. sh-USP5: sh-1#: 5′-GAATCGGAAGCAAGGACATTG-3′; sh-2#: 5′-CAGTCTTCCTGGAAGCGTTGA-3′; and sh-3#: 5′-TCGGTGTTCTCGAACTGACTT-3′. sh-VHL: sh-1#: 5′-GCCGAATGAACCGTCAGTT-3′; sh-2#: 5′-CAGCATGCCCTGTTCAAT-3′; and sh-3#: 5′-GATCGCGAAGAGTTGTGTTT-3′. Flag-tagged CYP1B1 (Gene ID: 1545; vector: pGMLV), Flag-HIF2α (Gene ID: 2034; vector: pGMLV), Flag-USP5 (Gene ID: 7375; vector: pGMLV), Flag-USP37 (Gene ID: 57695; vector: pGMLV), Flag-USP7 (Gene ID: 7874; vector: pGMLV), Flag-USP9X (Gene ID: 8239; vector: pGMLV), and Flag-USP28 (Gene ID: 57646; vector: pGMLV) were sourced from Genomeditech. GST-tagged VHL (Gene ID: 7428; vector: pGEX-4T-1), GST-tagged HIF2α (Gene ID: 2034; vector: pGEX-4T-1), and GST-tagged USP5 (Gene ID: 7375; vector: pGEX-4T-1) were procured from Tsingke Biotechnology (Beijing, China). HA-tagged CYP1B1 (Gene ID: 1545; vector: pCMV) and His-tagged VHL (Gene ID: 7428; vector: pGMLV) were also designed and synthesized by Tsingke Biotechnology. The efficiency of transfection was rigorously validated by quantitative real-time PCR (RT‒qPCR) and western blotting. After transduction, the cells were subjected to puromycin selection to establish stable cell lines for subsequent experimentation.

### Cell counting kit-8 (CCK-8) and EdU assays

The cells were seeded in 96-well plates at a concentration of 2000 cells per well in medium supplemented with 10 % FBS. After an overnight incubation period, the adherent cells were subjected to various treatments. Cell viability was evaluated following the CCK-8 protocol provided by Yeasen Biotechnology (Shanghai, China). For the EdU assay, the cells were cultivated in 12-well plates at a density of 2 × 10^4^ cells per well. Thereafter, the cells were fixed with a 4 % paraformaldehyde solution for 20 minutes at ambient temperature. Hoechst reagent and Apollo staining solution from RiboBio (Guangzhou, China) were used for staining. The percentage of EdU-positive cells was determined using a fluorescence microscope (Olympus IX71, Tokyo, Japan); five randomly selected fields were analysed for each sample.

### Transwell assay

Cell migration capacity was assessed via the Transwell migration assay. The cells were initially plated into 6-well plates at a density of 2 × 10^5^ cells per well. Once the cells reached 90 % confluency, 3 × 10^4^ cells per well in serum-free medium were added to the upper chamber of the Transwell system, while the lower chamber was filled with 500 µl of medium supplemented with 10 % FBS. After a 24-h incubation at 37°C in a controlled environment with 5 % CO_2_, the cells that had migrated to the lower surface of the membrane were fixed with 4 % paraformaldehyde. These cells were then stained with 0.1 % crystal violet for 60 minutes. The migration of the cells was quantified by counting the number of cells in five randomly selected fields under a microscope.

### Tube formation assay

Matrigel (ABW, Shanghai, China) was meticulously dispensed at a volume of 200 μl per well in a 48-well plate and incubated at 37°C for 30 minutes to induce polymerization. HUVECs were pretreated with either DMSO or PROTAC CYP1B1 degrader-1 (PCD-1) (MedChemExpress, Monmouth Junction, USA), a derivative of α-naphthoflavone chimaera designed to target CYP1B1 for degradation [20], for a period of 12 h. These cells were then seeded onto Matrigel-precoated wells at a density of 3 × 10^6^ cells per well and incubated at 37°C for 2.5 h. Subsequently, the cells were labelled with 2 μM calcein-AM (Beyotime, Shanghai, China) for 30 min at 37°C. Fluorescence microscopy imaging was conducted using an Olympus IX71 microscope (Tokyo, Japan) to visualize and document the cellular dynamics.

### Chick chorioallantoic membrane (CAM) assay

The antiangiogenic efficacy of PCD-1 was assessed via the CAM assay. Pathogen-free fertilized chicken eggs were procured from Boehringer Ingelheim Biology Company (Beijing, China). The eggs were incubated at 38.5°C with 5 % CO_2_ and 50 % humidity. After a 15-day incubation period, a sterile incision of approximately 1.5 cm in diameter was made in the eggshell to expose the CAM. A Teflon O-ring was then gently placed onto the CAM, and PCD-1 was applied within the ring. The window was sealed with sterile tape, and the eggs were returned to the incubator for an additional 72 h. Subsequently, the number of blood vessels was manually counted via a stereomicroscope (Leica M165 C, Germany).

### Flow cytometry analysis of cell apoptosis

Following transfection or drug treatment, the cells were harvested and subjected to a double-staining protocol with PI and Annexin V (Yeasen Biotechnology, Shanghai, China). After staining, the cells were incubated in the dark at room temperature for 10–15 minutes. The flow cytometry data were then collected via a CytoFlex cytometer (Beckman Coulter, USA), and analysis was performed via FlowJo V10 software.

### RT‒qPCR and RNA sequencing (RNA‒seq)

Total cellular RNA was meticulously extracted with TRIzol reagent (Servicebio Technology, Wuhan, China). The Hifair II 1st Strand cDNA Synthesis Kit (Yeasen Biotechnology, Shanghai, China) was used to synthesize first-strand cDNA according to the manufacturer's guidelines. Quantitative PCR analysis was conducted in accordance with the SYBR Green Master Mix protocol on a StepOne PCR instrument (Applied Biosystems, Foster, CA, USA), with GAPDH serving as the endogenous control for normalizing the mRNA expression levels. The primer sequences are presented in **Supplementary Table 1**. To assess the differences in the mRNA expression profiles between sunitinib-sensitive and resistant xenograft solid tumours, we conducted RNA sequencing. Total RNA was extracted from both types of tumours for the construction of RNA libraries, which were then subjected to next-generation sequencing to reveal the underlying transcriptomic profiles.

### Western blotting and coimmunoprecipitation (Co-IP)

Western blotting analysis was conducted in accordance with established methodologies [21,22]. Details of the primary and secondary antibodies used are provided in **Supplementary Table 2**. Co-IP assays were performed following the protocol supplied by Biolinkedin (Biolinkedin, Shanghai, China). Treated cells were lysed on ice for 30 minutes in cell lysis buffer supplemented with 1 % protease inhibitor and 1 % phosphatase inhibitor (Boster, Wuhan, China). The cell lysates were incubated with antibodies overnight at 4°C with continuous rotation to allow immunocomplex formation. Subsequently, precooled magnetic beads were added to the immunocomplexes and incubated for 2 h at room temperature. After three washes with wash buffer, the beads were collected and mixed with SDS‒PAGE sample loading buffer. The antigen‒antibody complexes that were bound to the beads were then denatured by boiling and subjected to western blotting.

### GST pull-down assays

Recombinant GST-tagged and Flag-tagged proteins were expressed in BL21 (Rosetta) bacterial cells and subsequently purified via a protein purification kit from Biolinkedin (Shanghai, China) according to the manufacturer's protocol. GST pull-down assays were executed in accordance with the instructions provided with the GST Protein Interaction Pull-Down Kit (Biolinkedin, Shanghai, China). In essence, the purified GST-tagged proteins were conjugated to glutathione-Sepharose beads. These beads, enriched with GST-tagged proteins, were incubated with His-tagged proteins in a pull-down buffer supplemented with PMSF. Post-incubation, the complexes were magnetically separated using a magnetic tube rack to collect beads with bound proteins, which were subsequently processed for western blotting.

### Immunofluorescence staining

Following transfection or drug treatment, the cells were plated onto glass coverslips in 12-well plates at a density of 1.5 × 10^5^ cells per well. The cells were fixed with a 4 % paraformaldehyde solution for 30 minutes at room temperature. After fixation, the samples were permeabilized with 0.5 % Triton X-100 (Solarbio, Beijing, China) for 15 minutes and subsequently rinsed with PBS. The cells were then blocked with goat serum (ZSGB-Bio, Beijing, China) before being incubated with primary antibodies overnight at 4°C. Thereafter, the cells were incubated with fluorescently labelled secondary antibodies. The coverslips were washed twice with PBS and then stained with DAPI for 10 minutes. Images were acquired using a confocal microscope (LSM880, Zeiss, Germany). Details on the primary and fluorescent secondary antibodies used are in **Supplementary Table 2**.

### Animal studies

All animal experiments conducted in this study were approved by the Institutional Animal Care and Use Committee of the First Affiliated Hospital of Shihezi University (A2024-339). Five-week-old female athymic nude mice and weighing 16–20 grams, were procured from SIPEIFU Biotechnology (Beijing, China). The inhibitory effect of PCD-1 on tumour angiogenesis was evaluated via the Matrigel plug assay. 786O cells (1 × 10^6^) were mixed with DMSO, PCD-1 (100 nM or 200 nM) or bevacizumab (0.5 nM), and subsequently combined with Matrigel at a 1:2 ratio in a total volume of 200 µl. The resulting suspension was subcutaneously injected into the dorsal region of nude mice. PCD-1 (2 mg/kg) or bevacizumab (0.5 mg/kg) was administered via intraperitoneal injection every other day. After a period of 14 days, the degree of vascularization within the Matrigel plug was quantified, and the tumours were harvested for immunohistochemistry (IHC) with antibodies against CD31 and VEGFA. To establish a subcutaneous tumour xenograft model, 2 × 10^6^ transfected ccRCC cells were inoculated subcutaneously into the mice. After 7 days to allow for tumour initiation and growth, the mice were randomly assigned to different groups. The tumour dimensions were assessed and recorded every 4 days. Upon completion of the 28-day study, the mice were euthanized in a humane manner, and the tumours were extracted, measured, and archived for subsequent analysis. The orthotopic xenograft model of ccRCC was generated as previously described [23,24]. In summary, 1 × 10^6^ SU-R 786O cells were injected into the left renal region of the mice. The mice were subsequently randomly divided into four groups and received the following treatments via oral (p.o.) administration or intraperitoneal (i.p.) injection: (1) DMSO, (2) sunitinib at a dose of 20 mg/kg, (3) PCD-1 at a dose of 2 mg/kg, and (4) a combination of sunitinib at 20 mg/kg and PCD-I at 2 mg/kg. After a 28-day treatment regimen, the mice were humanely euthanized, and the tumours were harvested for further analysis.

### Bioinformatics analysis

The mRNA expression data for CYP1B1 in ccRCC tissues and corresponding paracancerous tissues, along with clinical patient data, were obtained from The Cancer Genome Atlas (TCGA) database. The relationship between CYP1B1 expression and patient survival was evaluated via Kaplan‒Meier survival analysis. Differentially expressed genes between the high and low CYP1B1 groups were identified with criteria of a fold change greater than 2 and a p value <0.05. Functional enrichment analysis was then conducted to reveal the roles of the differentially expressed genes. We also utilized the UbiBrowser database to predict the deubiquitinating enzymes that target the HIF2α protein.

### Statistical analysis

The data are presented as the means ± standard deviations. Statistical comparisons between two groups were conducted via Student's t-test for normally distributed data, and the Wilcoxon test was applied for data with skewed distributions. Survival curves were analysed via the log-rank test. All the statistical analyses were performed via R version 4.3.0 and GraphPad Prism version 8. A p value of <0.05 was considered to indicate statistical significance.

## Results

### CYP1B1 is aberrantly express in SU-R RCC

**Fig. 1:**
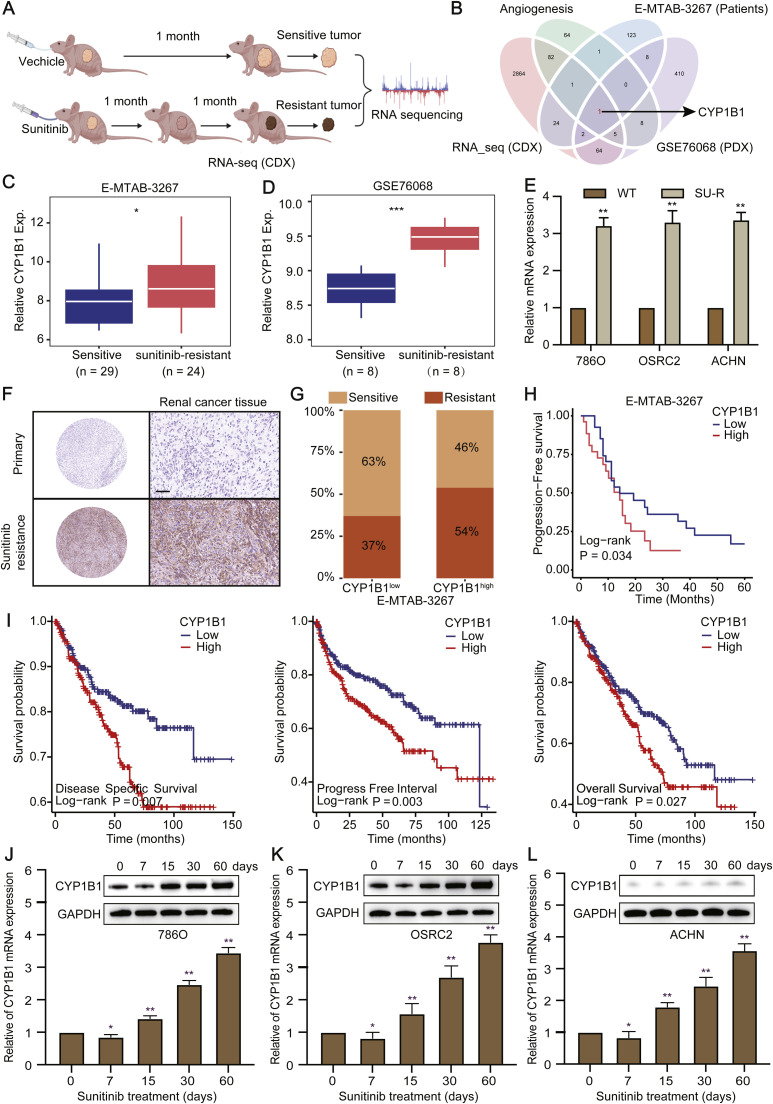
Elevated CYP1B1 expression in SU-R RCC. **A** Graphical representation of sunitinib-resistant CDX models and differential gene expression analysis between sunitinib-sensitive and sunitinib-resistant tumours via RNA-seq. **B** Screening of angiogenesis-related genes in the CDX model and two independent SU-R RCC datasets revealed CYP1B1 as a critical gene in sunitinib resistance. **C** and **D** Comparison of CYP1B1 expression in sunitinib-sensitive versus sunitinib-resistant tumours across the E-MTAB-3267 and GSE76068 datasets. **E** CYP1B1 mRNA levels in three pairs of wild-type (WT) and SU-R RCC cell lines. **F** CYP1B1 protein expression was analysed in primary and SU-R tumour tissues from 12 patients. Scale bar: 100 μm. **G** and **H** Correlations between CYP1B1 levels and sunitinib resistance and progression-free survival in SU-R RCC patients in the E-MTAB-3267 cohort. **I** Differences in overall survival, progression-free survival, and disease-specific survival between CYP1B1^high^ and CYP1B1^low^ patients in the TCGA cohort. **J**-**L** CYP1B1 induction by sunitinib (2.5 μM) over a 0–60-day period in three RCC cell lines, with mRNA and protein levels assessed at specified time points via RT‒qPCR and western blotting (ns, not significant; *, *p* < 0.05; **, *p* < 0.01).

To thoroughly elucidate the mechanisms underlying sunitinib resistance in RCC, we established SU-R cell-derived xenograft (CDX-R) models via sunitinib administration (Fig. 1A). Compared with WT 786O cells, 786O SU-R cells derived from these CDX models exhibited reduced sensitivity to sunitinib, as evidenced by an increased half-maximal inhibitory concentration (IC50) (**Figure S1A**). We performed RNA-seq analysis of three pairs of CDX samples to identify key genes and biological processes implicated in sunitinib resistance. The differentially expressed genes were subjected to functional enrichment analysis, which revealed that multiple vasculature-related processes, including angiogenesis and vascular development regulation, were significantly enriched in sunitinib resistance-related genes (**Figure S1B, C**). Consequently, we conducted a comparative analysis of various SU-R models, including CDX models, PDX models (GSE76068), and patient samples (E-MTAB-3267) (Fig. 1B), to identify potential angiogenesis-related genes responsible for sunitinib resistance. The screening results suggested that CYP1B1 may play a pivotal role in angiogenesis, as its expression was significantly elevated in both SU-R RCC cells and patient samples compared to controls (Fig. 1C–E).

Through an in-depth analysis of the TCGA database, we found that the CYP1B1 expression level was positively correlated with tumour stage, lymphatic metastasis, and metastatic stage (**Figure S1D-F**). To further validate the role of CYP1B1 in sunitinib resistance, we assessed CYP1B1 protein levels in RCC samples via IHC. Consistent with our previous findings, we observed that CYP1B1 protein levels were significantly elevated in tissues exhibiting acquired resistance to sunitinib compared to primary tumour tissues without acquired resistance (Fig. 1F, **S1H**). In the sunitinib treatment cohort (E-MTAB-3267), following treatment, patients with high CYP1B1 expression demonstrated worse progression-free survival than those with low CYP1B1 expression (Fig. 1G, H). Moreover disease-specific survival, progression-free interval, and overall survival were significantly greater in the low CYP1B1 expression group than in the high CYP1B1 expression group (Fig. 1I). Furthermore, we assessed the mRNA and protein levels of CYP1B1 at various time points during the induction of sunitinib resistance in RCC. As depicted in Fig. 1J,K, both CYP1B1 mRNA and protein expression progressively increased in 786O and OSRC2 ccRCC cells exposed to sunitinib for 60 days. However, in the ACHN papillary RCC cell line, the mRNA levels of CYP1B1 increased to a greater degree than the protein levels did, and the protein expression of CYP1B1 remained relatively low in ACHN cells (Fig. 1L).

### pVHL mediates the ubiquitination and subsequent degradation of CYP1B1

**Fig. 2:**
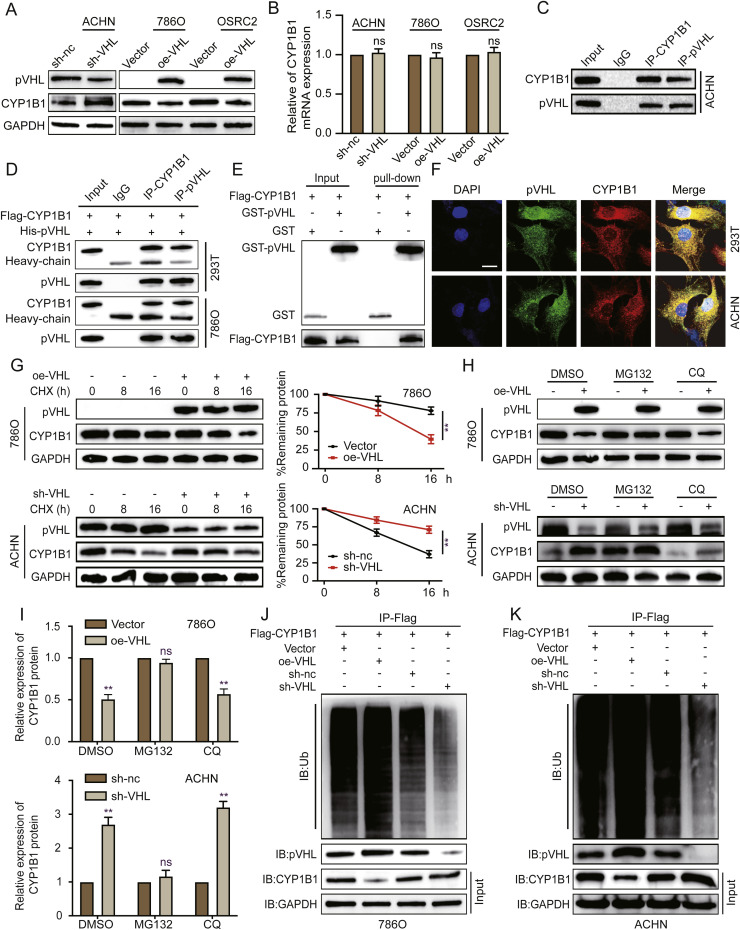
pVHL mediates the ubiquitination and degradation of CYP1B1. **A** and **B** Assessment of CYP1B1 mRNA and protein levels in ACHN cells with stable VHL knockdown and 786O and OSRC2 cells with stable VHL overexpression via western blotting and RT‒qPCR. **C** Coimmunoprecipitation of ACHN cell whole-cell lysates with CYP1B1, pVHL or IgG antibodies, followed by western blotting with the respective antibodies. **D** 293T and 786O cells were transfected with Flag-CYP1B1 and His-pVHL plasmids. Coimmunoprecipitation with CYP1B1, pVHL or IgG antibodies was followed by western blotting with the indicated antibodies. **E** GST affinity-isolation assays with purified tagged pVHL and CYP1B1 proteins, followed by western blotting analysis. **F** Cotransfection of 293T and ACHN cells with VHL and CYP1B1 overexpression plasmids and subsequent immunofluorescence staining with anti-pVHL and anti-CYP1B1 antibodies. Scale bar: 10 μm. **G** Protein synthesis inhibition via CHX (10 μM) in 786O cells with VHL overexpression and ACHN cells with VHL knockdown; CYP1B1 protein levels were assessed by western blotting at specified time points. **H** and **I** Treatment of 786O and ACHN cells with VHL overexpression or knockdown plasmids, followed by exposure to DMSO, chloroquine (CQ, 10 μM), or MG132 (20 μM) for 8 hours; CYP1B1 protein levels were analysed by western blotting. **J** and **K** Immunoprecipitation of cells with anti-Flag CYP1B1 antibodies, followed by western blotting with the indicated antibodies (ns, not significant; **, *p* < 0.01).

Given that the protein expression level of CYP1B1 was significantly greater in the VHL-mutated RCC cell line than in the VHL wild-type ACHN cell line, we hypothesized that VHL might affect CYP1B1 expression. To test this hypothesis, we assessed the effect of VHL knockdown or overexpression on CYP1B1 expression in RCC cells and found that CYP1B1 protein levels significantly increased following VHL knockdown in ACHN cells and considerably decreased after VHL overexpression in 786O and OSRC2 cells (Fig. 2A). Interestingly, RT‒qPCR data revealed that the alteration of VHL expression had no effect on the mRNA expression levels of CYP1B1 (Fig. 2B). This discrepancy between the protein and mRNA levels of CYP1B1 suggests that VHL may regulate CYP1B1 levels by affecting the stability of the CYP1B1 protein rather than by affecting its transcription. Therefore, we investigated whether pVHL interacts with CYP1B1. The Co-IP results revealed that both exogenous and endogenous CYP1B1 form a protein complex with pVHL (Fig. 2C,D). The GST pull-down experiment confirmed that CYP1B1 directly binds to pVHL proteins (Fig. 2E). Additionally, immunofluorescence staining revealed that pVHL and CYP1B1 colocalize in the cytoplasm (Fig. 2F).

To further elucidate the mechanism by which VHL regulates CYP1B1, we treated cells with cycloheximide (CHX) and observed that VHL overexpression significantly decreased the half-life of the CYP1B1 protein in VHL-mutated 786O and OSRC2 cells. Conversely, VHL depletion in VHL wild-type ACHN cells led to the stabilization of the CYP1B1 protein (Fig. 2G, **S2A**). Considering that pVHL is an E3 ligase, we hypothesized that it directly ubiquitinates CYP1B1, targeting it for degradation. To test this hypothesis, we assessed the effects of proteasome and lysosomal inhibitors on CYP1B1 protein expression regulated by VHL [20,25]. Western blotting confirmed our hypothesis, as the proteasome inhibitor MG132 reversed the decrease in the CYP1B1 protein level induced by VHL overexpression in 786O and OSRC2 cells and reversed the increase in the CYP1B1 protein level induced by VHL depletion in ACHN cells (Fig. 2H, I and **S2B**). Subsequent Co-IP experiments revealed that the ubiquitination level of CYP1B1 increased upon VHL overexpression and decreased when VHL was depleted in RCC cells (Fig. 2J, K, and **S2C**). Together, these findings indicate that VHL negatively regulates CYP1B1 protein stability through the ubiquitin‒proteasome pathway.

### CYP1B1 regulates the sensitivity of ccRCC cells to sunitinib

**Fig. 3:**
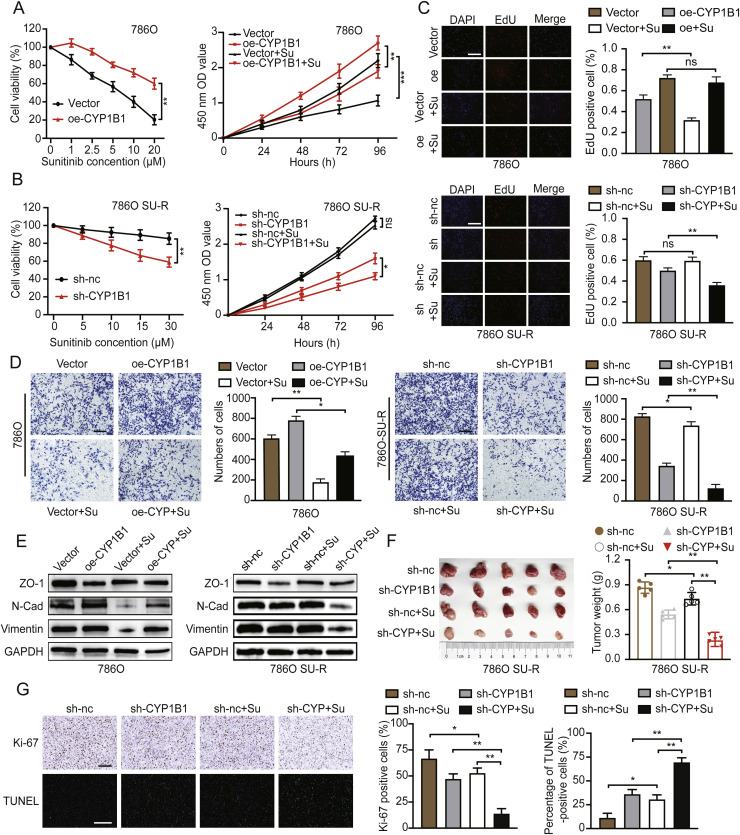
CYP1B1 modulates sunitinib sensitivity in ccRCC. **A** and **B** Establishment of stable CYP1B1-overexpressing WT 786O cells and CYP1B1-knockdown SU-R 786O cells. The cells were treated with DMSO or sunitinib, and cell viability was assessed via CCK-8 assays. **C and D** Evaluation of cell proliferation and migration in the indicated groups via EdU incorporation and Transwell assays. Scale bar: 50 μm. **E** Expression of epithelial‒mesenchymal transition markers in the different treatment groups. **F** Nude mice bearing SU-R xenografts with stable CYP1B1 knockdown were treated with vehicle control or sunitinib (20 mg/kg, p.o.) for 4 weeks. The tumour size was monitored every four days, and the tumours were weighed and photographed at the endpoint. **G** IHC staining of Ki-67 in tumours and a TUNEL assay for apoptosis analysis in the four treatment groups. Scale bar: 100 μm (ns, not significant; *, *p* < 0.05; **, *p* < 0.01; ***, *p* < 0.001).

To investigate the biological function of CYP1B1 in modulating sunitinib resistance, we overexpressed CYP1B1 in WT ccRCC cells and knocked it down in SU-R ccRCC cells (**Figure S3A and S3B**). The overexpression of CYP1B1 increased the sunitinib resistance of WT 786O and OSRC2 cells (Fig. 3A and **S3C**), whereas the knockdown of CYP1B1 increased the sensitivity of SU-R 786O and OSRC2 cells to sunitinib (Fig. 3B and **S3D**). Additionally, EdU proliferation assays confirmed that increased CYP1B1 expression mitigated the suppressive effects of sunitinib on the proliferation of WT 786O cells. Conversely, CYP1B1 knockdown intensified the inhibitory effects of sunitinib on the proliferation of SU-R 786O cells (Fig. 3C). Next, we assessed the impact of changes in CYP1B1 expression on the migratory capacity of RCC cells via Transwell assays. We found that the overexpression of CYP1B1 significantly promoted the migration of WT ccRCC cells and mitigated the inhibitory effect of sunitinib on the migration of those cells. Conversely, knockdown of CYP1B1 suppressed the migration of SU-R ccRCC cells and increased the inhibitory effect of sunitinib on their migration (Fig. 3D and **S3E**). Furthermore, we assessed changes in the levels of markers associated with the epithelial‒mesenchymal transition. Compared with the control group, the group with overexpression of CYP1B1 exhibited notable increases in the levels of N-cadherin and vimentin and a decrease in ZO-1 expression upon sunitinib treatment. Conversely, in SU-R RCC cells, CYP1B1 depletion led to reduced levels of N-cadherin and vimentin and increased ZO-1 expression following sunitinib treatment (Fig. 3E and **S3F**). To validate the impact of CYP1B1 on sunitinib sensitivity in vivo, we established xenograft models using control and CYP1B1-knockdown SU-R 786O cells. The mice were randomly grouped and treated with either DMSO or sunitinib. The volume of tumours derived from SU-R 786O cells was not significantly different between the sunitinib group and the DMSO group. However, knockdown of CYP1B1 reduced the tumour volume and significantly enhanced the inhibitory effect of sunitinib on tumour growth (Fig. 3F and **S3G**). The effects of CYP1B1 on the proliferation and apoptosis indices of SU-R 786O xenografts were further confirmed by IHC staining (Fig. 3G). Collectively, these results suggest that CYP1B1 knockdown increases the sensitivity of ccRCC to sunitinib treatment.

### CYP1B1 induces VM in tumour cells by promoting HIF2α/VEGFA signalling

**Fig. 4:**
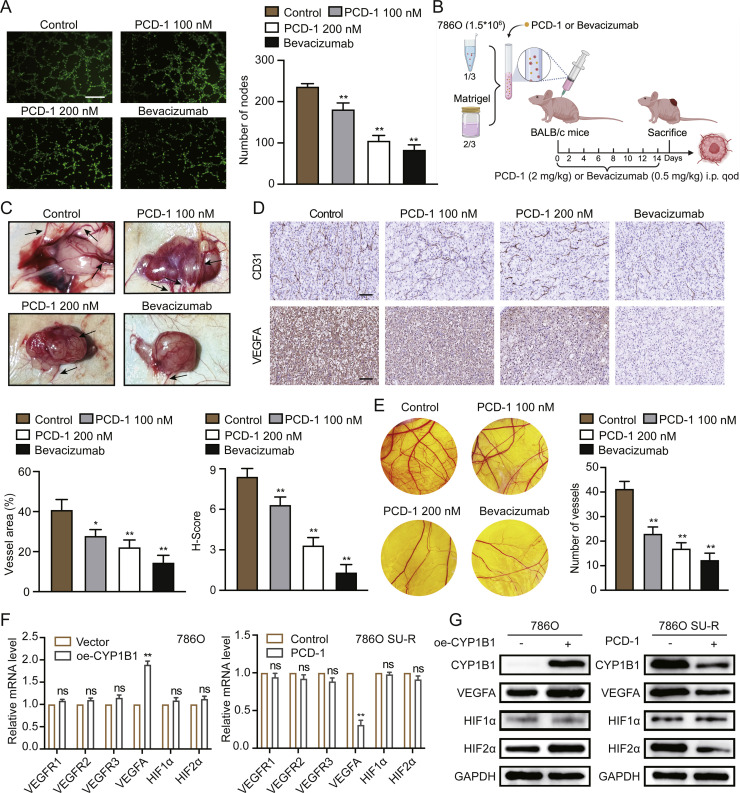
CYP1B1 promotes VM and enhances HIF2α/VEGFA signalling in ccRCC. **A** Impact of the CYP1B1 inhibitor PCD-1 on the tube formation of HUVECs. Scale bar: 50 μm. **B** and **C** Graphical representation and representative images of Matrigel plug assays following treatment with DMSO, PCD-1, or bevacizumab. **D** IHC staining of VEGFA and CD31 in Matrigel plugs from the indicated groups, with quantification of vessel area and VEGFA-positive cells. Scale bar: 100 μm. **E** Inhibition of new vessel formation by PCD-1 in the CAM assay. **F** and **G** RT‒qPCR and western blotting analysis of HIF1α, HIF2α, VEGFA, VEGFR1, VEGFR2, and VEGFR3 expression in ccRCC cells treated with DMSO and PCD-1 (100 nM) for 48 hours or transfected with vector or CYP1B1 expression plasmid (ns, not significant; *, *p* < 0.05; **, *p* < 0.01).

Analysis of previous RNA sequencing data revealed a close association between sunitinib resistance and angiogenesis, particularly the regulation of vascular development, in RCC (**Figure S1C**). Given that CYP1B1 is a key molecule in angiogenic signalling pathways, we hypothesized that it may play a significant role in regulating VM and tumour angiogenesis. To test this hypothesis, we investigated the antiangiogenic effects of CYP1B1 both in vitro and in vivo. As shown in Figure 4A, 200 nM PCD-1 potently inhibited the tube formation of HUVECs; this effect was comparable to that of bevacizumab (50 ng/ml). To further explore the antiangiogenic activity of CYP1B1 in vivo, we conducted Matrigel plug and CAM assays. We mixed Matrigel with RCC 786O cells, with or without PCD-1, and subcutaneously implanted the Matrigel plugs into the mice (Fig. 4B). PCD-1 significantly blocked angiogenesis in the tumour plugs (Fig. 4C), as confirmed by CD31 and VEGFA IHC staining (Fig. 4D). Furthermore, PCD-1 markedly inhibited neoangiogenesis and microvessel development in the CAM assay (Fig. 4E). ccRCC is often characterized by inactivating mutations in the VHL gene, which lead to HIFα-mediated VEGFA production and the development of highly vascularized tumours [[26], [27], [28]]. To investigate the role of CYP1B1 in this pathway, we compared the mRNA levels of components of the HIFα/VEGFA/VEGFR signalling pathway between the control and PCD-1 groups and between the vector and CYP1B1 overexpression groups. As shown in Fig. 4F and **S4A**, PCD-1 potently repressed VEGFA mRNA expression in SU-R 786O cells, while the mRNA expression levels of HIFs and VEGFRs remained similar between the groups. Similarly, CYP1B1 overexpression in 786O cells significantly upregulated VEGFA mRNA expression but had no effect on HIF and VEGFR mRNA expression levels. However, western blot analysis revealed that CYP1B1 not only increased VEGFA protein expression but also significantly increased HIF2α protein expression (Fig. 4G and **S4B**). Taken together, these results suggest that CYP1B1 may promote RCC angiogenesis by promoting HIF2α/VEGFA signalling.

### CYP1B1 regulates the stability of the HIF2α protein

**Fig. 5:**
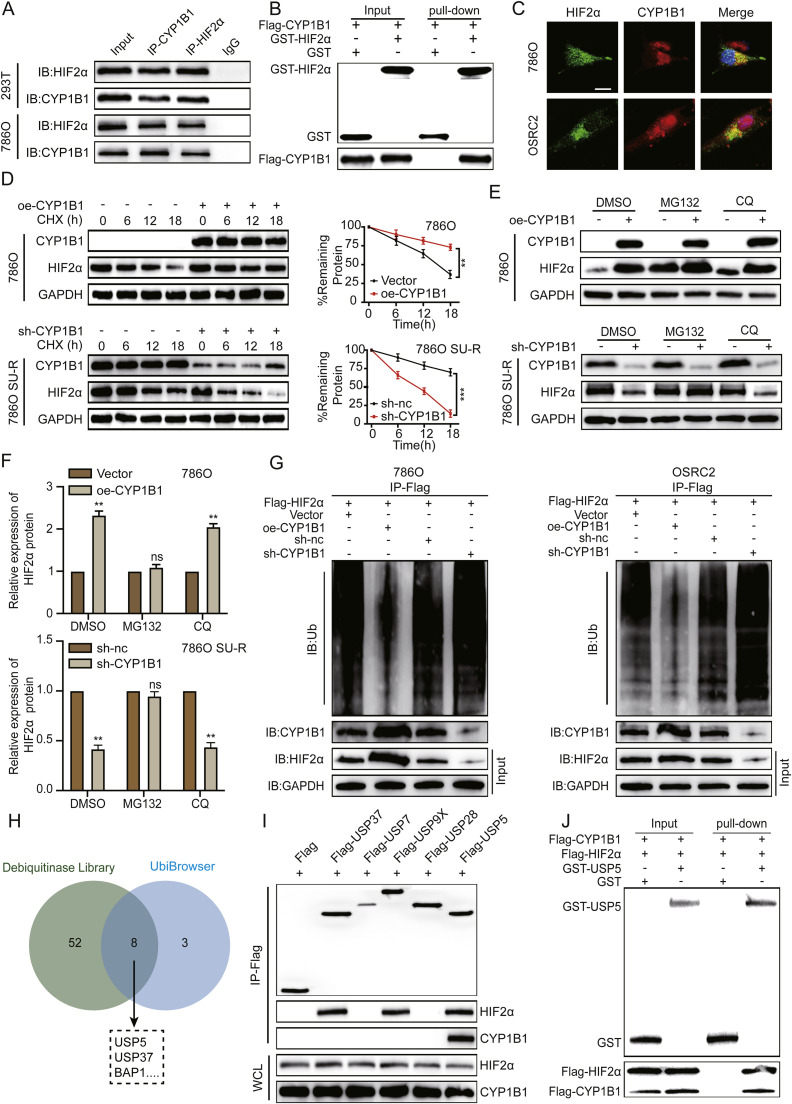
CYP1B1 regulates HIF2α protein stability through the ubiquitin–proteasome pathway. **A** Immunoprecipitation of 293T and 786O cell lysates with CYP1B1 or HIF2α antibodies, followed by western blotting with the indicated antibodies. **B** The purified tagged proteins were subjected to GST pull-down assays with the indicated antibodies. **C** Confocal microscopy analysis of HIF2α and CYP1B1 colocalization in 786O and OSRC2 cells. Scale bar: 50 μm. **D** Protein synthesis inhibition in 786O cells overexpressing CYP1B1 and SU-R 786O cells with CYP1B1 knockdown via CHX (10 μM); HIF2α protein levels were determined by western blotting at specified time points. **E** and **F** Treatment of 786O cells overexpressing CYP1B1 and SU-R 786O cells with CYP1B1 knockdown with DMSO, chloroquine (10 μM), or MG132 (20 μM) for 8 hours; HIF2α protein levels were analysed by western blotting. **G** Immunoprecipitation of the indicated cell lysates with anti-Flag antibodies, followed by western blotting with the indicated antibodies. **H** Venn diagram illustrating potential deubiquitinating enzymes that regulate the degradation of ubiquitinated HIF2α. **I** Co-IP assays in 786O cells were performed to assess the interaction between the five deubiquitinating enzymes and HIF2α. **J** The purified tagged proteins were subjected to GST pull-down assays with the indicated antibodies (ns, not significant; **, *p* < 0.01; ***, *p* < 0.001).

On the basis of the above results, we hypothesized that CYP1B1 regulates HIF2α protein stability through direct protein‒protein interactions. To test this hypothesis, we performed Co-IP and GST pull-down assays, which demonstrated that CYP1B1 can directly bind to HIF2α (Fig. 5A, B). Immunofluorescence assays were also conducted on 786O and OSRC2 cells to examine the subcellular localization of CYP1B1 and its interaction with HIF2α. Confocal microscopy revealed that CYP1B1 was highly expressed and colocalized with HIF2α in the cytoplasm (Fig. 5C). Furthermore, we performed nuclear and cytoplasmic fractionation of 786O and OSRC2 cells and then performed Co-IP experiments. Notably, the CYP1B1–HIF2α protein complex was detected primarily in the cytoplasm, which is the predominant subcellular compartment for HIF2α protein degradation (**Figure S5A**). After treating ccRCC cells stably overexpressing CYP1B1 and SU-R ccRCC cells with CYP1B1 knockdown with CHX, we observed significant changes in HIF2α protein stability. Compared with that in the control group, the half-life of HIF2α was considerably extended in 786O and OSRC2 cells overexpressing CYP1B1, whereas SU-R RCC cells with CYP1B1 knockdown exhibited a markedly reduced HIF2α protein half-life (Fig. 5D and **S5B**). To further elucidate the mechanisms underlying the CYP1B1-mediated regulation of HIF2α protein stability, ccRCC cells were exposed to proteasome inhibitors (MG132) and lysosomal inhibitors (chloroquine). As shown in Fig. 5E and F, only the proteasome inhibitor MG132 reversed the increases in the HIF2α protein induced by CYP1B1 overexpression or the decreases induced by CYP1B1 knockdown. Further assays revealed that the overexpression of CYP1B1 in 786O and OSRC2 cells decreased the level of ubiquitinated HIF2α (Fig. 5G). These findings indicate that CYP1B1 may increase HIF2α protein stabilization via the ubiquitin–proteasome pathway.

To elucidate the mechanism by which CYP1B1 regulates HIF2α protein ubiquitination, we utilized a deubiquitinase library, which was reported by Hong et al. [23] for predicting potential deubiquitinases that bind to HIF2α, in conjunction with the online prediction tool Ubibrowser. This screening process yielded eight candidate deubiquitinase genes (Fig. 5H). Three of these deubiquitinases have been previously reported to be unable to bind to HIF2α [23,29]. Additionally, Hong's research demonstrated that two deubiquitinases (USP37 and USP9X) can stabilize HIF2α through deubiquitination [23]. On the basis of these findings, we conducted Co-IP assays to validate the interactions among HIF2α, CYP1B1, and the five deubiquitinases. The western blotting results revealed that only USP5 interacts with both CYP1B1 and HIF2α (Fig. 5I). Additionally, a GST pull-down experiment confirmed that USP5 directly binds to both the CYP1B1 protein and the HIF2α protein (Fig. 5J).

### CYP1B1 promotes HIF2α protein stabilization by promoting the interaction between USP5 and HIF2α

**Fig. 6:**
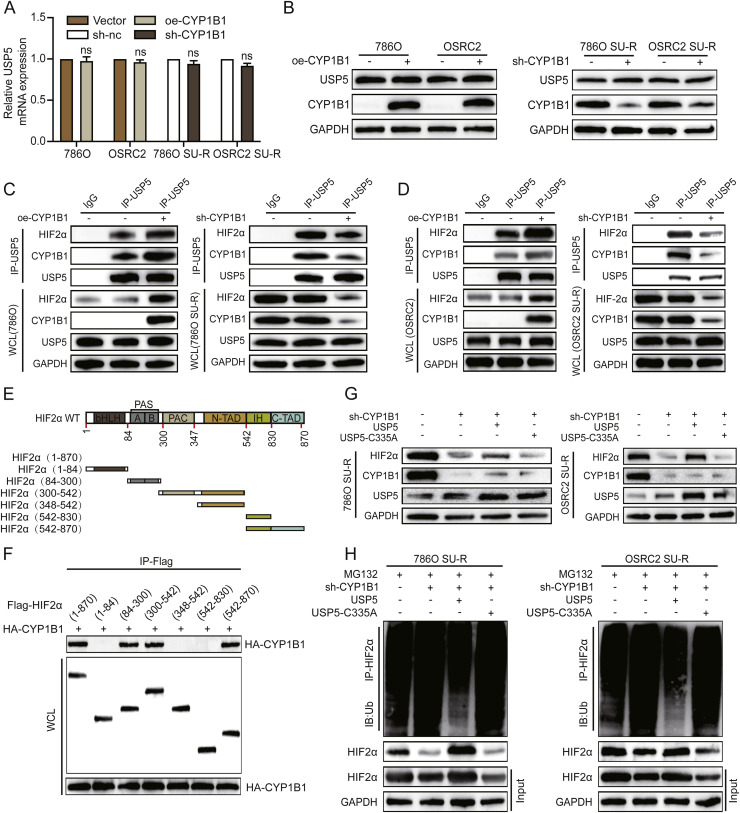
CYP1B1 promotes HIF2α protein stabilization by enhancing the interaction between USP5 and HIF2α. **A** and **B** The mRNA and protein levels of CYP1B1 in RCC cells were analysed when the cells were subjected to CYP1B1 knockdown or overexpression. **C** and **D** WT and SU-R RCC cells were transfected with a CYP1B1 expression plasmid or shRNA for knockdown. Co-IP with IgG or USP5 antibody was followed by western blotting with the indicated antibodies. **E** and **F** Co-IP assays were conducted on 293T cells transfected with HA-labelled CYP1B1, Flag-labelled HIF2α and structural domain plasmids (Flag-HIF2α-bHLH, Flag-HIF2α-PAS, Flag-HIF2α-NTAD, Flag-HIF2α-IH, Flag-HIF2α-PAC—NTAD, and Flag-HIF2α-IH—CTAD). **G** SU-R RCC cells were transfected with the CYP1B1 knockdown plasmid alone or in combination with the USP5 wild-type or mutant USP5-C335A plasmid. Western blotting was performed with the indicated antibodies. **H** These cell lysates were immunoprecipitated with IgG or HIF2α antibodies and then subjected to western blotting with the indicated antibodies (ns, no significance).

USP5 is a member of the ubiquitin-specific protease family of deubiquitinases, which play critical roles in ubiquitin-mediated signalling and protein quality control [30,31]. In the TCGA-KIRC cohort, USP5 expression was significantly increased in ccRCC tumour tissues, and its mRNA levels were strongly correlated with lymph node and distant metastasis (**Figure S6A**). Interestingly, neither CYP1B1 knockdown nor CYP1B1 overexpression affected USP5 mRNA or protein expression levels (Fig. 6A and B). However, western blotting revealed that the interaction between HIF2α and USP5 was enhanced by CYP1B1 overexpression in ccRCC cells but suppressed by CYP1B1 depletion in SU-R ccRCC cells (Fig. 6C, D). To identify the structural domains required for the HIF2α‒CYP1B1 interaction, we coexpressed a series of truncated HIF2α constructs with HA‒CYP1B1 in 293T cells and performed Co-IP assays (Fig. 6E). Our analysis revealed that the PAS, PAC, and C-TAD domains of HIF2α were sufficient for interaction with CYP1B1 (Fig. 6F).

Given that the enzymatically inactive mutant of USP5 (USP5-C335A) cannot remove ubiquitin chains from target proteins [32], we conducted rescue western blotting assays to determine whether the deubiquitination activity of USP5 is essential for CYP1B1-mediated HIF2α protein expression. As shown in Fig. 6G, only wild-type USP5, not the USP5-C335A mutant, reversed the downregulation of the HIF2α protein caused by CYP1B1 depletion in SU-R ccRCC cells. Conversely, the upregulation of HIF2α induced by CYP1B1 overexpression was reversed by USP5 knockdown (**Figure S6B**). Consistent with our previous findings, CYP1B1 depletion promoted HIF2α ubiquitination, and this effect was attenuated by wild-type USP5 but not by USP5-C335A (Fig. 6H).

### CYP1B1 inhibition suppresses SU-R RCC progression

**Fig. 7:**
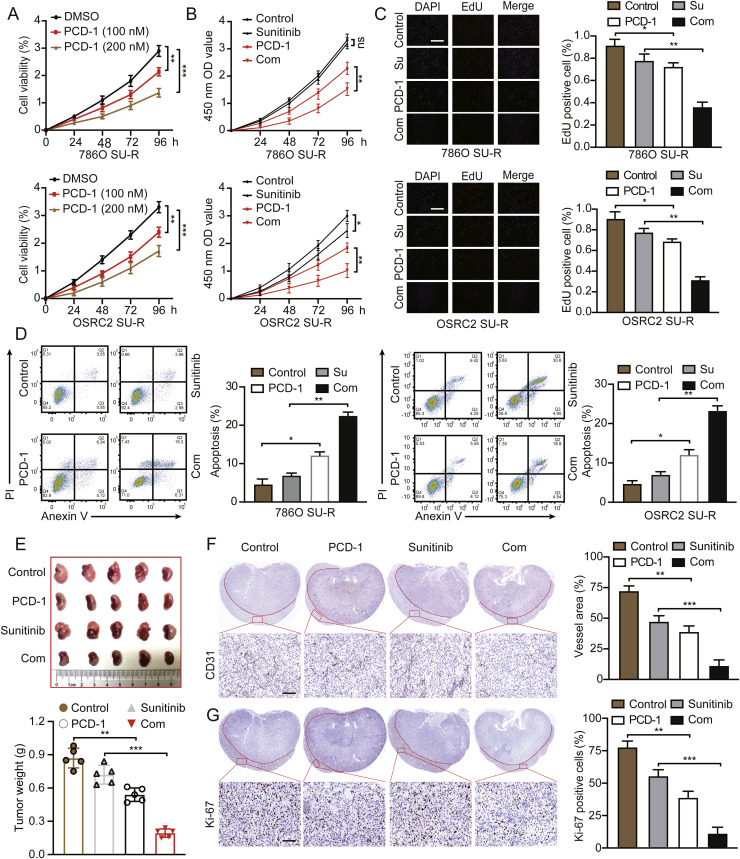
Inhibition of CYP1B1 impedes the progression of SU-R ccRCC. **A** Assessment of the viability of SU-R ccRCC cells treated with DMSO or PCD-1 (100 nM or 200 nM) at various time points via CCK-8 assays. **B-D** SU-R ccRCC cells were treated with DMSO, sunitinib (10 μM), PCD-1 (100 nM), or their combination for 0–96 hours. Cell viability was evaluated via CCK-8 assays, and cell proliferation and apoptosis were assessed via EdU incorporation and flow cytometry assays. Scale bar: 50 μm. **E** Establishment and treatment of an orthotopic xenograft model using SU-R 786O cells (1 × 10^6^). The mice were treated with vehicle, sunitinib (20 mg/kg), PCD-1 (2 mg/kg), or their combination for 28 days, after which they were sacrificed, and the tumour weights were measured. **F** and **G** IHC staining of Ki-67 and CD31 expression levels in tumours from the four treatment groups. Scale bar: 50 μm (ns, not significant; *, *p* < 0.05; **, *p* < 0.01; ***, *p* < 0.001).

**Fig. 8:**
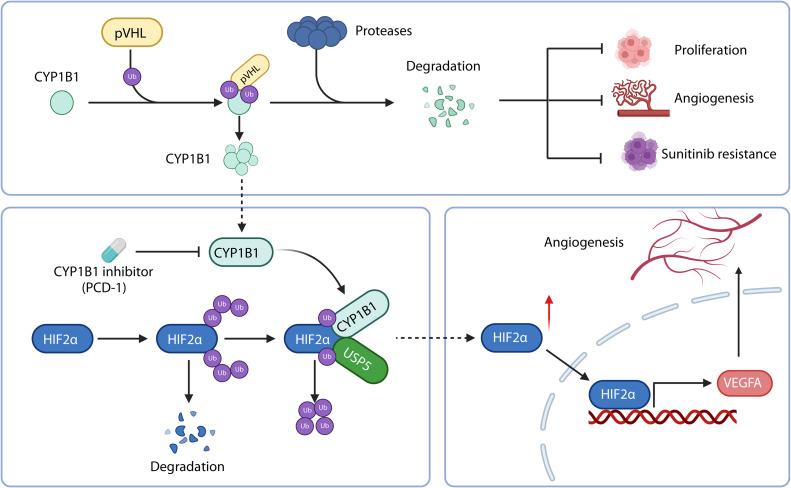
Schematic representation of the mechanistic role of CYP1B1 in ccRCC: upregulation of CYP1B1 in VHL-mutated ccRCC promotes ccRCC progression and angiogenesis. CYP1B1 interacts directly with the HIF2α protein, impeding its degradation in a USP5-dependent process, thereby sustaining the HIF2α/VEGFA signalling axis and facilitating the emergence of sunitinib resistance.

Activation of the HIF2α/VEGFA axis decreases the effectiveness of anticancer therapy and has been identified as a mechanism related to sunitinib desensitization [26,27,33]. Consistent with these findings, we found that HIF2α and VEGFA levels were significantly upregulated in SU-R ccRCC cells compared with WT cells (**Figure S7A**). Hence, we treated WT and SU-R ccRCC cells with PCD-1, and the results suggested that inhibition of CYP1B1 suppressed the protein expression of HIF2α in a time- and dose-dependent manner (**Figure S7B**). Moreover, PCD-1 significantly decreased the proportion of viable SU-R 786O and OSRC2 cells (Fig. 7A). Importantly, CCK-8 and EdU assays revealed that PCD-1 significantly increased the sensitivity of SU-R RCC cells to sunitinib (Fig. 7B, C). The results of the apoptosis assay confirmed the above findings (Fig. 7D). Western blotting revealed increases in the levels of the proapoptotic proteins BAX and caspase 3 and decreases in the levels of HIF2α and VEGFA in response to the combination treatment with sunitinib and PCD-1 (**Figure S7C**). To assess the therapeutic efficacy of PCD-1 more accurately in a setting that closely resembles the biological and clinical context of RCC, we established an orthotopic xenograft model. Consistent with the in vitro results, combination treatment with PCD-1 noticeably reversed the resistance of SU-R RCC cells to sunitinib (Fig. 7E). The combination treatment groups exhibited a significant reduction in the number of Ki-67-positive cells, tumor vessel area, and HIF-2α expression (Fig. 7F, G and **S7D**). In contrast, USP5 expression remained largely unchanged across all groups (**Figure S7E**). Taken together, these findings indicate that CYP1B1 is a potential therapeutic target for treating ccRCC and that PCD-1 in combination with sunitinib synergistically attenuate acquired sunitinib resistance (Fig. 8).

## Discussion

Sunitinib has been a standard treatment for ccRCC for more than a decade [34,35]. However, the mechanisms underlying both intrinsic and acquired sunitinib resistance remain incompletely understood. The majority of ccRCC cases are characterized by VHL gene inactivation, leading to persistent HIF2α activation, which drives the expression of angiogenic factors and promotes tumour angiogenesis [28]. Increasing evidence suggests that cancer cells can form vessel-like structures and generate blood vessels independently of endothelial cells, a process strongly associated with resistance to TKIs in ccRCC [36,37]. Notably, we found that CYP1B1 expression was significantly increased in ccRCC cells with inactivated VHL gene, and revealed the potential mechanism that pVHL degrades CYP1B1 through the ubiquitination pathway. Furthermore, we revealed a previously unrecognized feed-forward regulatory loop involving VHL deficiency, CYP1B1 accumulation, and HIF2α stabilization that perpetuates sunitinib resistance. Through validation in multiple models, including CDX models, PDX models, and clinical specimens, we systematically demonstrated that CYP1B1 overexpression is a critical molecular determinant of resistance. Mechanistically, CYP1B1 orchestrates HIF2α stabilization via USP5-mediated deubiquitination, creating a self-reinforcing signalling axis that sustains angiogenic signalling despite anti-VEGF therapy.

Cytochrome P450 enzymes play essential roles in various biological processes and human diseases because of their involvement in the metabolism of a broad range of substrates [[38], [39], [40]]. Previous studies have shown that CYP1B1 is capable of metabolizing and either activating or inactivating therapeutic drugs [[41], [42], [43]]. Xue et al. reported that CYP1B1 plays a pivotal role in driving PARP inhibitor resistance through distinct molecular mechanisms in the A2780-OlaR cell line [41]. In addition, CYP1B1 has been shown to decrease the efficacy of anti-PD-1 therapy in colorectal cancer by preventing ferroptosis through the degradation of ACSL4 [17]. In our study, we identified a previously unrecognized pathway in which CYP1B1 stabilizes HIF-2α through USP5-mediated deubiquitination, forming a self-reinforcing signaling axis. Notably, knockdown of CYP1B1 significantly enhanced the sensitivity of sunitinib-resistant 786O and OSRC2 cells to sunitinib, an effect that was further validated in vivo using xenograft models.

Hypoxia-inducible factors are well-established regulators of gene expression involved in angiogenesis, glycolysis, metastasis, and treatment resistance [44]. While HIF1α is ubiquitously expressed, HIF2α expression is more restricted, particularly in highly vascularized organs or hypoxic tissues [45]. Recent clinical studies have demonstrated that belzutifan, a small-molecule HIF2α inhibitor, provides significant clinical benefits in the treatment of VHL-associated tumours [[46], [47], [48]]. The combination of belzutifan with TKIs has shown promising antitumour activity in pretreated ccRCC patients [49]. Our study revealed that CYP1B1 orchestrates HIF2α stabilization via USP5-mediated deubiquitination. Furthermore, we revealed a reciprocal relationship between VHL loss and CYP1B1 accumulation, wherein VHL deficiency promotes CYP1B1 stabilization, which in turn prevents the proteasomal degradation of HIF2α through USP5 recruitment. This dual regulatory mechanism not only perpetuates HIF2α signalling but also provides a molecular basis for the limited efficacy of sunitinib in VHL-mutant tumours. Importantly, the clinical relevance of this axis is underscored by our findings that CYP1B1 knockdown restored sunitinib sensitivity in vivo and that combined CYP1B1 inhibition and sunitinib treatment yielded tumour suppression effects superior to those of monotherapy.

Nonetheless, several limitations of this study should be acknowledged. First, the role of CYP1B1 in sunitinib resistance requires further validation in prospective clinical cohorts. Second, the correlation between CYP1B1 and HIF-2α expression should be further confirmed in sunitinib-resistant ccRCC tissue samples. In addition, while the deubiquitinase activity of USP5 is essential for CYP1B1-mediated stabilization of HIF-2α, it remains unclear whether the interaction between CYP1B1 and USP5 directly affects this enzymatic activity. This question will be a key focus of our future investigations.

In summary, our findings highlight the central role of ubiquitin pathway dysregulation in ccRCC progression. While pVHL is well recognized as an E3 ligase adaptor for HIFα degradation, its newly identified role in regulating CYP1B1 turnover adds another layer of complexity to the role of the ubiquitin‒proteasome system in therapeutic resistance. This expanded understanding may facilitate biomarker development and supports the potential value of CYP1B1 as a therapeutic target for sunitinib-resistant ccRCC patients.

## CRediT authorship contribution statement

**Ke Ma:** Writing – original draft, Validation. **Qinyu Li:** Validation, Data curation. **Yi Zhang:** Validation, Software. **Jiuyi Wang:** Investigation. **Wei Jia:** Formal analysis. **Jihong Liu:** Methodology. **Bo Liu:** Resources. **Qiang Li:** Writing – review & editing, Visualization. **Qinzhang Wang:** Supervision, Funding acquisition. **Kai Zeng:** Writing – review & editing, Conceptualization.

## Declaration of competing interest

The authors declare that they have no known competing financial interests or personal relationships that could have appeared to influence the work reported in this paper.

## Appendix Supplementary materials

Image, application 1
