# Supplementary material for: CYP1B1 promotes angiogenesis and sunitinib resistance in clear cell renal cell carcinoma via USP5-mediated HIF2α deubiquitination
Source: Neoplasia. 2025 May 27;66:101186. doi: 10.1016/j.neo.2025.101186 (PMC12158534; doi:10.1016/j.neo.2025.101186)
Supplement: Supplementary file 1 [file mmc1.pdf]

CYP1B1 promotes angiogenesis and sunitinib resistance in clear cell renal cell carcinoma through USP5-mediated HIF2α deubiquitination

Ke Ma[a, †], Qinyu Li[b, †], Yi Zhang[a, †], Jiuyi Wang[a], Wei Jia[c], Jihong Liu[d], Bo Liu[b], Qiang Li[a, *], Qinzhang Wang[a, *], Kai Zeng[a,d *]

[a]Department of Urology, the First Affiliated Hospital of Shihezi University, Shihezi, Xinjiang, China
[b]Department of Oncology, Tongji Hospital, Tongji Medical College, Huazhong University of Science and Technology, Wuhan, Hubei, China
[c]Department of Pathology, the First Affiliated Hospital of Shihezi University, Shihezi, Xinjiang, China
[d]Department of Urology, Tongji Hospital, Tongji Medical College, Huazhong University of Science and Technology, Wuhan, Hubei, China

† These authors contributed to this work equally and shared first authorship.

Corresponding to:

Kai Zeng, zengkai@shzu.edu.cn

Qinzhang Wang, wqz1969@sina.com

Qiang Li, liqiangbl123@sina.com

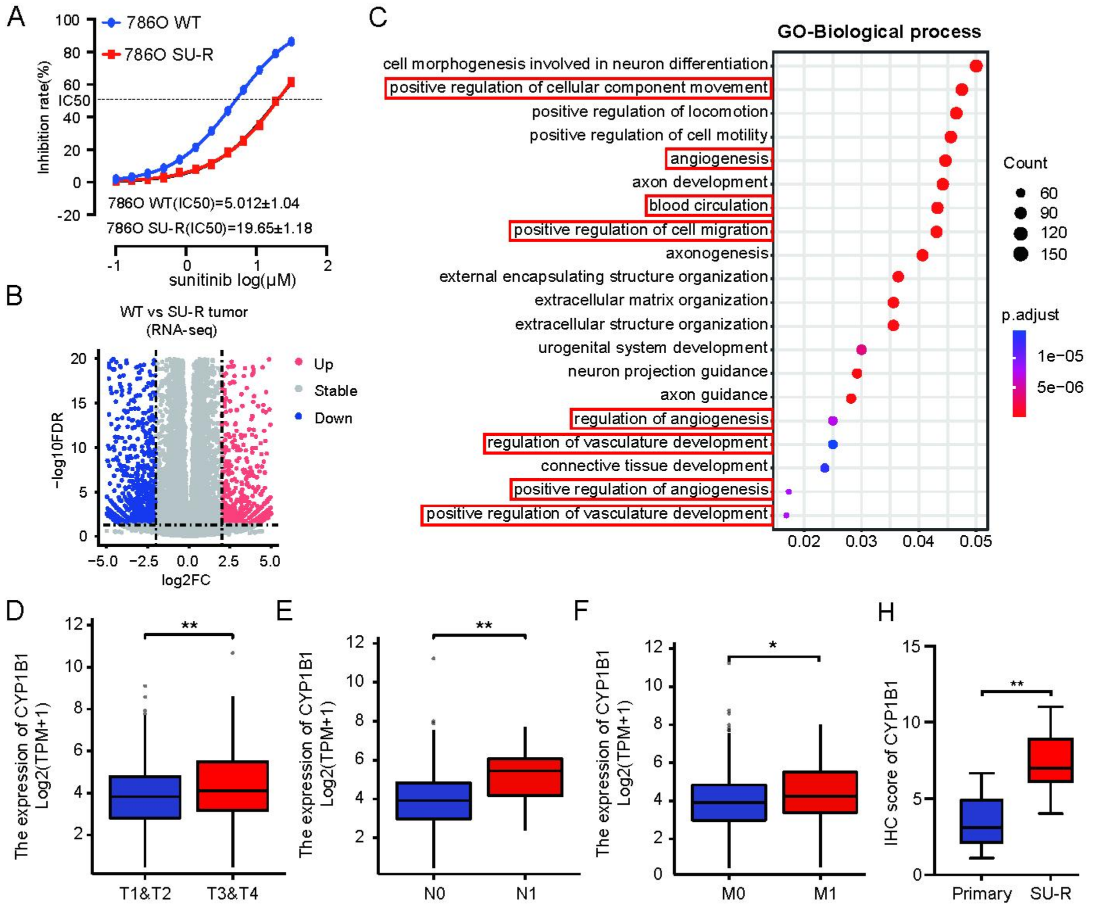

Figure S1. A WT or SU-R 786O cells were treated with varying concentrations of sunitinib for 48 h, and cell viability was assessed using CCK-8 assays. **B** Volcano plot depicting differential gene expression between sunitinib-sensitive and -resistant tumors. **C** GO enrichment analysis of differentially expressed genes in sensitive versus resistant CDX tumors. **D-F** Correlation analysis between CYP1B1 expression and various clinicopathological factors. (*, $p < 0.05$; **, $p < 0.01$).

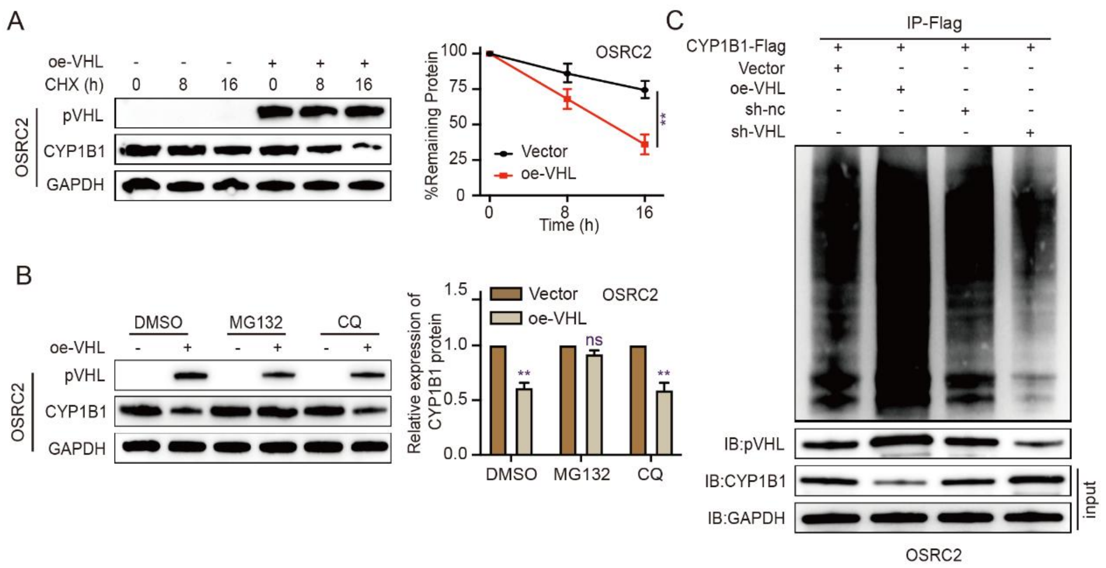

Figure S2. A OSRC2 cells were transfected with a VHL overexpression plasmid and subsequently treated with CHX (10 μM) to inhibit protein synthesis. CYP1B1 protein levels were determined by western blotting at the specified time points. **B** OSRC2 cells overexpressing VHL were treated with DMSO, chloroquine (10 μM), or MG132 (20 μM) for 8 hours, and CYP1B1 protein levels were detected and analyzed by western blotting. **C** OSRC2 cells were transfected with VHL overexpression or knockdown plasmids, followed by immunoprecipitation with anti-Flag CYP1B1 antibodies and western blotting with the indicated antibodies.

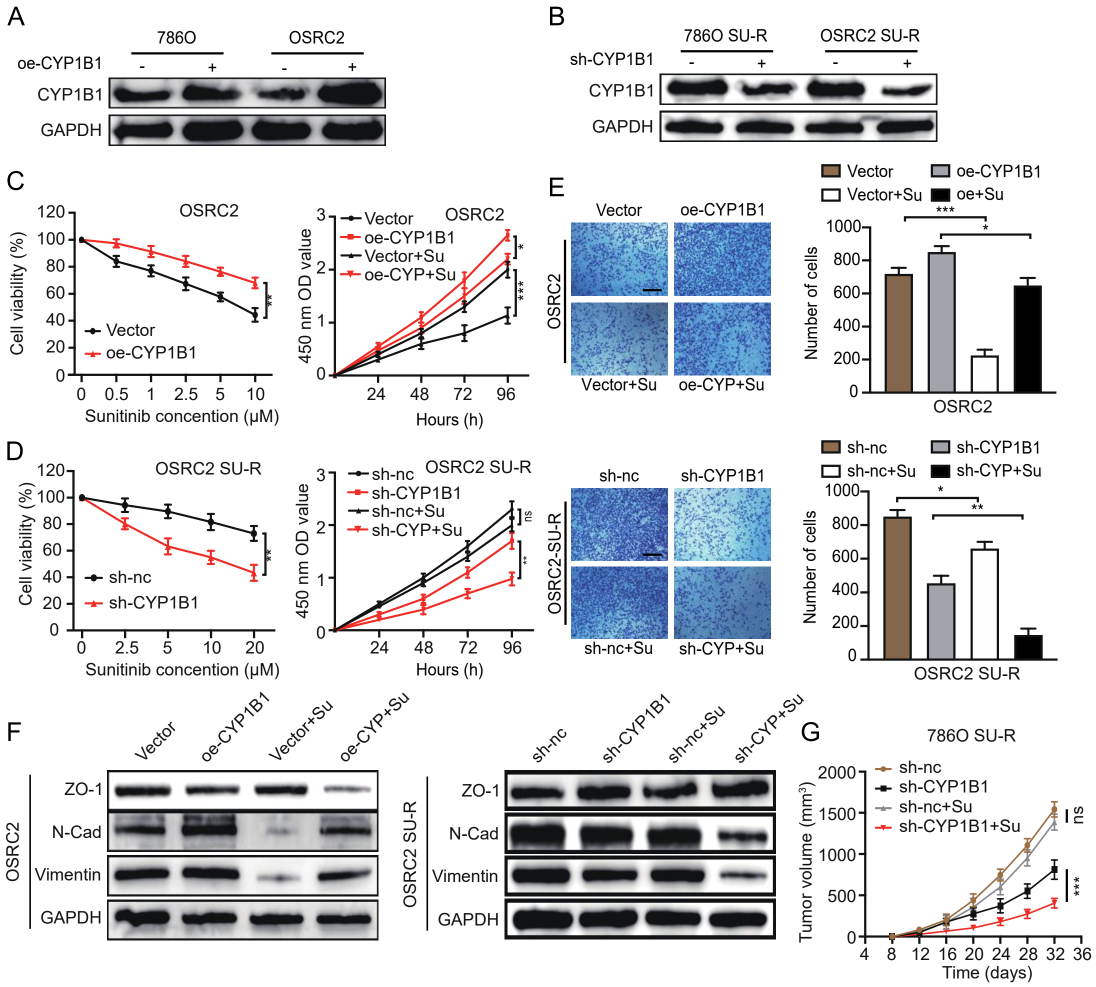

Figure S3. A and B Validation of CYP1B1 overexpression or knockdown effects in OSRC2 cells by western blotting. **C** and **D** Establishment of WT OSRC2 cells stably overexpressing CYP1B1 and SU-R OSRC2 cells stably knockdown for CYP1B1. Cells were treated with DMSO or sunitinib, and cell viability was assessed using CCK-8 assays. **E** Assessment of cell migration in the indicated groups using Transwell assays. Scale bar: 50 μm. **F** Changes in epithelial-mesenchymal transition markers between the different treatment groups. **G** Growth curve of subcutaneous tumors in mice from the four treatment groups.

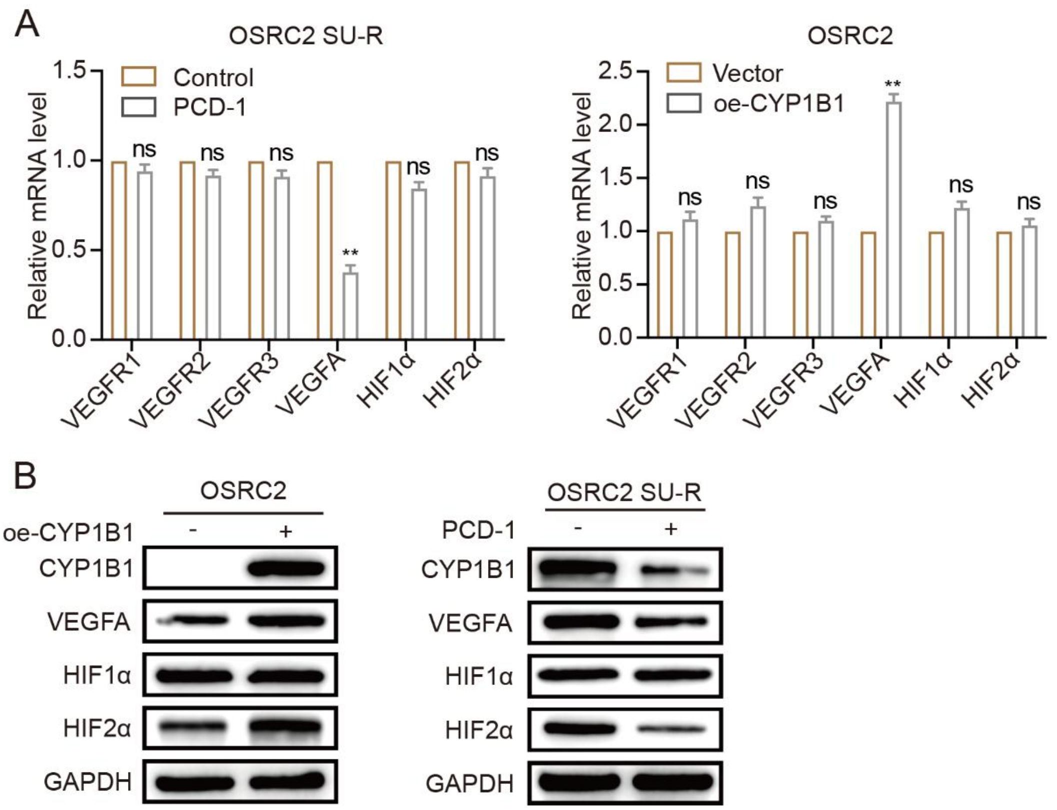

Figure S4. RT-qPCR and western blotting analysis of HIF1α, HIF2α, VEGFA, VEGFR1, VEGFR2, and VEGFR3 expression in OSRC2 cells treated with DMSO and PCD-1 (100 nM) for 48 hours or transfected with vector and CYP1B1 expression plasmid.

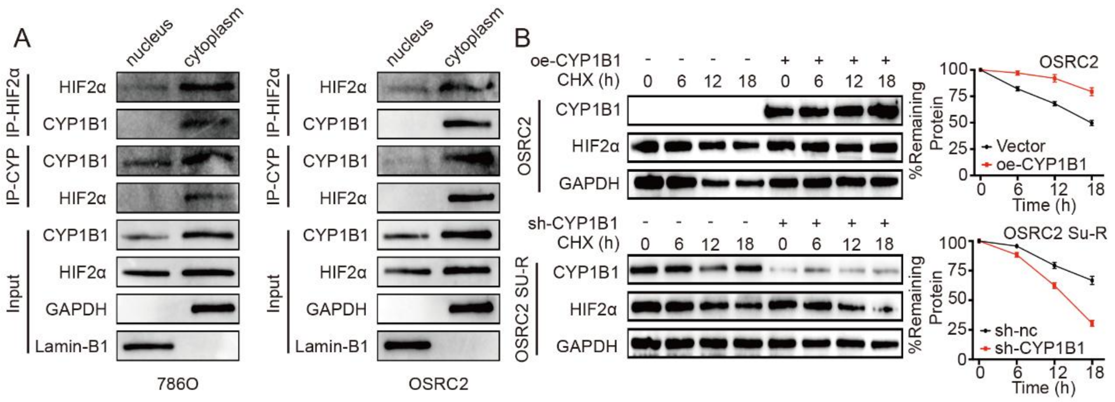

Figure S5. A Co-IP assays were conducted using nuclear and cytosolic extracts from 786O and OSRC2 cells to investigate protein interactions between CYP1B1 and HIF2α. **B** OSRC2 cells were transfected with a CYP1B1 overexpression plasmid, and SU-R OSRC2 cells were transfected with a CYP1B1 knockdown plasmid. Following transfection, cells were treated with CHX (10 μM) to inhibit protein synthesis, and HIF2α protein levels were determined by western blotting at specified time points.

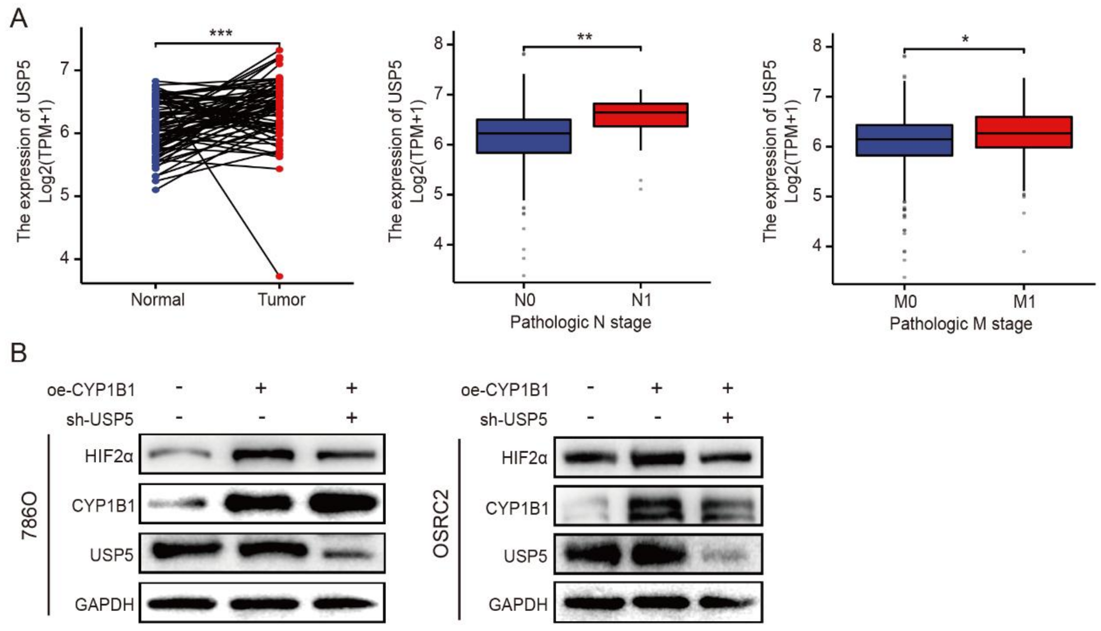

Figure S6. A Examination of the relationship between CYP1B1 expression levels and various clinicopathological factors in TCGA-KIRC cohort. **B** 786O and OSRC2 cells were transfected with a CYP1B1 knockdown plasmid alone or in combination with a USP5 expression plasmid for 48 hours. Western blotting was performed to analyze the protein levels with the indicated antibodies.

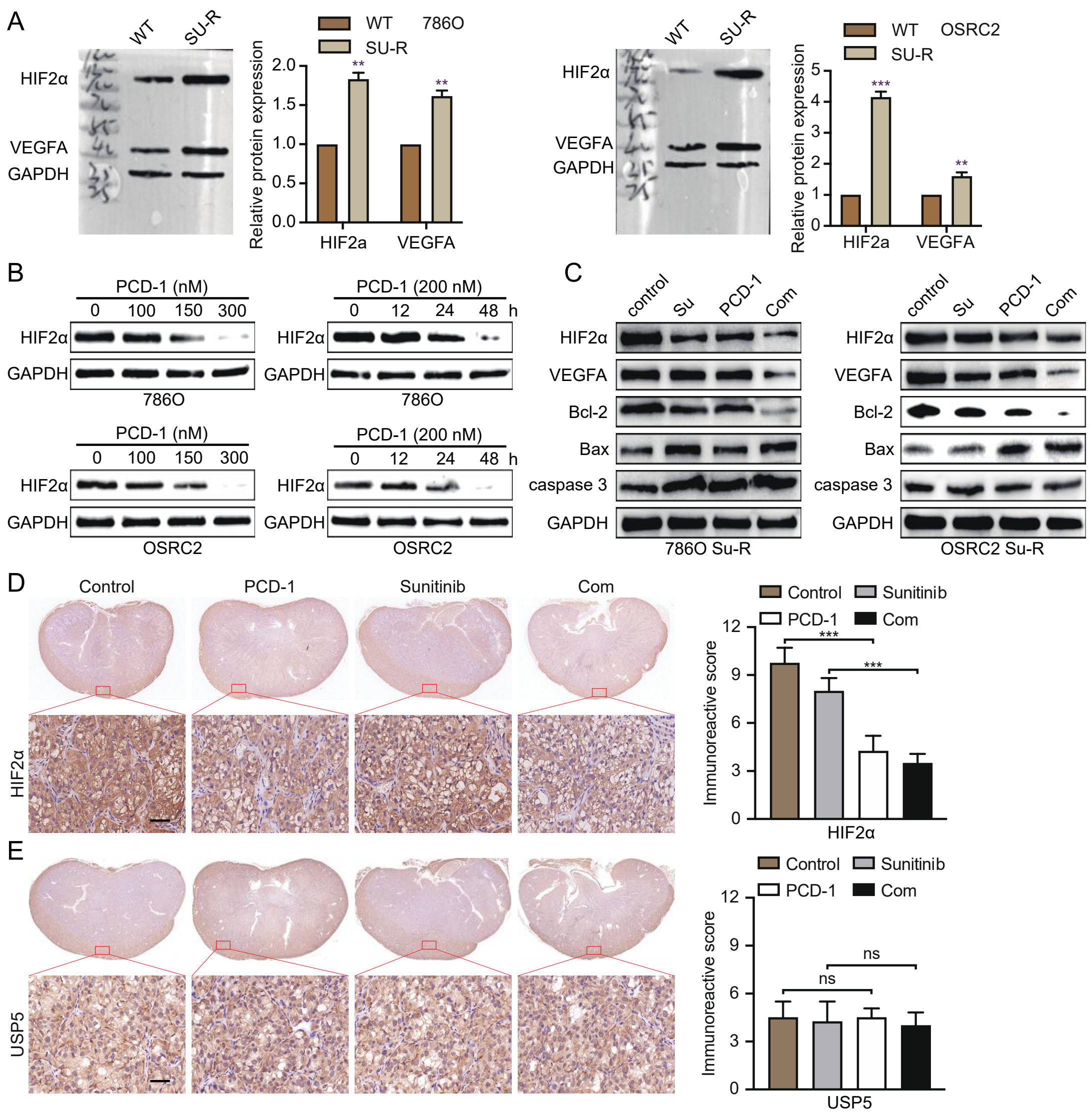

Figure S7. **A** Western blotting was used to determine HIF2α and VEGFA protein levels in WT and SU-R ccRCC cells. **B** 786O and OSRC2 cells were treated with PCD-1 at the indicated concentrations for 48 hours, or for the indicated time points. **C** SU-R ccRCC cells were treated with DMSO, sunitinib (10 μM), PCD-1 (100 nM), or in combination for 0–96 hours. Changes in apoptosis markers were assessed between the four treatment groups. **D** and **E** IHC staining of HIF2α and USP5 expression levels in orthotopic tumors from the four indicated groups. Scale bar: 50 μm (ns, not significant; ***, $p < 0.001$).

Supplementary Table 1 Sequence of primers for qRT-PCR analysis

**Primers used in q-PCR analysis**		
GAPDH	Forward primer	CGCTCTCTGCTCCTCCTGTTC
	Reverse primer	ATCCGTTGACTCCGACCTTCAC
USP5	Forward primer	GGCAACTCCTGCTACATAAACAG
	Reverse primer	CGAATAATATTAGCTTGTGGGACTG
CYP1B1	Reverse primer	GAAGGTGAAGGTCGGAGT
	Reverse primer	GAAGATGGTGATGGGATTTC
HIF1α	Forward primer	TCTGTGGACCTGTCGGTGATGG
	Reverse primer	CCTTATCAAGATGCGAACTCACA
HIF2α	Forward primer	CGGAGGTGTTCTATGAGCTGG
	Reverse primer	AGCTTGTGTGTTCGCAGGAA
VEGFA	Forward primer	GAGGGCAGAATCATCACGAAG
	Reverse primer	TGTGCTGTAGGAAGCTCATCTCTC
VEGFR1	Forward primer	TGGCCATCACTAAGGAGCACTCC
	Reverse primer	GGAACTGCTGATGGCCACTGTG
VEGFR2	Forward primer	CGGACAGTGGTATGGTTCTTGC
	Reverse primer	GTGGTGTCTGTGTCATCGGAGTG
VEGFR3	Forward primer	GTACATGCCAACGACACAGG
	Reverse primer	TGATGAATGGCTGCTCAAAG

Supplementary Table 2 Primary antibodies used in the study

Antibody	Company (Cat. No.)	Working dilutions
Bax	Abcam (ab32503)	WB:1/5000
Bcl-2	Abcam (ab32124)	WB:1/1000
caspase3	Abcam (ab32351)	WB:1/5000
CD31	Abcam (ab28364)	IHC:1/50
CYP1B1	Abcam (ab185954)	WB:1/2000, IHC:1/250
CYP1B1	Abcam (ab185954)	IF:1/250
GAPDH	Proteintech (60004-1-Ig)	WB:1/5000
HIF1α	Affinity (BF8002)	WB:1/1000
HIF2α	CST (71565S)	WB:1/1000, IF:1/100
HIF2α	Abcam (ab109616)	IHC:1/100， IF:1/100
USP5	Abcam (ab244290)	IHC:1/50,WB:1/1000
Ki-67	Abcam (ab16667)	IHC:1/200
N-cadherin	Abcam (ab98952)	WB:1/1000
Ubiquitin	Abcam (ab7254)	WB:1/1000
VEGFA	Proteintech (19003-1-AP)	WB:1/1000, IHC:1/100
Vimentin	Abcam (ab8978)	WB:1/1000
VHL	CST (2738)	WB:1/1000, IF:1/100
ZO-1	Abcam (ab190085)	WB:1/1000
Mouse anti Flag	Abclone (AE005)	WB:1/2000
Mouse anti His	Abclone (AE003)	WB:1/2000
Rabbit anti Flag	Abclone (AE063)	WB:1/2000
Rabbit anti GST	Abclone (AE006)	WB:1/1500
Rabbit anti HA	Abclone (AE105)	WB:1/2000
**Secondary Antibody**		
Goat Anti-Rabbit	Abclone (AS014)	WB:1/5000
Goat Anti-Mouse	Abclone (AS003)	WB:1/5000
Cy3 Goat Anti-Rabbit	Servicebio (GB21303)	IF:1/500
Cy3 Donkey Anti-Mouse	Servicebio (GB21401)	IF:1/500
FITC Goat Anti-Mouse	Servicebio (GB22301)	IF:1/500
FITC Goat Anti-Rabbit	Servicebio (GB22303)	IF:1/500
